# Two Tier Hox Collinearity Mediates Vertebrate Axial Patterning

**DOI:** 10.3389/fcell.2018.00102

**Published:** 2018-09-04

**Authors:** Antony J. Durston

**Affiliations:** Faculty of Science, Institute of Biology Leiden, Leiden University, Leiden, Netherlands

**Keywords:** hox collinearity, gastrulation, axial patterning, Xenopus laevis, antisense RNA

## Abstract

A two tier mechanism mediates Hox collinearity. Besides the familiar collinear chromatin modification within each Hox cluster (nanocollinearity), there is also a macrocollinearity tier. Individual Hox clusters and individual cells are coordinated and synchronized to generate multiscale (macro and nano) collinearity in the early vertebrate embryo. Macro-collinearity is mediated by three non-cell autonomous Hox–Hox interactions. These mediate temporal collinearity in early NOM (non-organizer mesoderm), time space translation where temporal collinearity is translated to spatial collinearity along the early embryo’s main body axis and neural transformation, where Hox expression is copied monospecifically from NOM mesoderm to overlying neurectoderm in the late gastrula. Unlike nanocollinearity, which is Hox cluster restricted, axial macrocollinearity extends into the head and EAD domains, thus covering the whole embryonic anterior-posterior (A-P) axis. EAD: extreme anterior domain, the only A-P axial domain anterior to the head. The whole time space translation mechanism interacts with A-P signaling pathways via “decision points,” separating different domains on the axis.

## Introduction

Hox Collinearity – the ordered temporal and spatial expression of Hox genes, in sequences matching their genomic order in the Hox clusters, is a problem that has fascinated and intrigued biologists since it was discovered. It is central for emergent areas in contemporary medicine – targeted destruction of specific cancers, stem cell therapy and *in vitro* organoid culture (Hasty et al., 1991; Shah and Sukumar, 2010; Howell and Wells, 2011; Lippmann et al., 2015). Elucidating the mechanisms regulating this phenomenon is of prime importance. In this article I review the collinearity problem and reach a very surprising conclusion that contributes to solving it.

## Hox Collinearity in Evolution

The fact that bilaterian Hox genes are often found in genomic clusters showing evolutionarily conserved collinearity- that the spatial sequence in which these genes are usually expressed along the early embryo’s anterior-posterior (A-P) body axis and sometimes also the temporal sequence in which they are expressed often both match their genomic sequence (Lewis, 1978; Monteiro and Ferrier, 2006; Duboule, 2007) obviously has something to do with genesis of the bilaterian bodyplan. Both of these collinearities are now extremely well documented in a variety of organisms and their existence has been generally accepted for > ∼20 years. It is also now the consensus that temporal collinearity leads to spatial collinearity during early vertebrate development (Duboule, 1994; Wacker et al., 2004; Durston and Zhu, 2015; Deschamps and Duboule, 2017).

How collinearity works is a mystery. The nature of the connection between spatial and temporal collinearities particularly is extraordinarily elusive. A striking fact is that the same spatial sequence of Hox genes is expressed throughout many bilateria, even in larvacean *Oikopleura* that has its non-clustered Hox genes dispersed throughout the genome (Seo et al., 2004 see also Monteiro and Ferrier, 2006; Duboule, 2007). This indicates an aspect of collinearity that is not Hox cluster dependent- an inference that is not at all surprising in the light of what follows. This is the first piece of evidence indicating a multi-tiered Hox collinearity mechanism. Studying *Drosophila*, the best understood bilaterian, has not been helpful. Here, the conserved spatially collinear Hox sequence is established by an upstream hierarchy of segmentation genes where different individual Hox genes are first activated differently by combinations of gap and pair-rule genes (Ingham and Martinez-Arias, 1986; Irish et al., 1989). This is most likely a specialist mechanism in *Drosophila* that has little relevance for collinearity and patterning in other organisms except that the original spatial Hox sequence is somehow still conserved.

In vertebrates, where Hox collinearity begins concurrently with segmentation during gastrulation at the time when the A-P axis of the bodyplan is first being generated, rather than later than the start of A-P patterning as in Drosophila, there is more chance that Hox collinearity plays a role in generating the body’s A-P pattern. Details follow.

## A Two Tier Mechanism for Vertebrate Hox Collinearity

Below, I summarize evidence pointing to a novel development in understanding Hox collinearity control. In vertebrates this is regulated by two layers of control, not one as has been supposed previously (Spitz et al., 2003, 2005; Soshnikova and Duboule, 2009; Noordermeer et al., 2014; Fabre et al., 2015). This control mechanism has a specific role in generating the vertebrate body plan.

### The Current Accepted Wisdom

Hox chromatin shows collinearity related phenomena. During temporal collinearity, global enhancers sequentially switch Hox genes from repressed to activated expression (Spitz et al., 2003, 2005); Hox genes transit sequentially from an inactive polycomb compartment to an active trithorax compartment (Soshnikova and Duboule, 2009; Noordermeer et al., 2014); Hox clusters deform progressively as they are expressed (Fabre et al., 2015). These findings are current accepted wisdom.

### Macro-Collinearity

Hox collinearity is multiscale. It not only happens over 100 nm. in an individual Hox cluster, it also happens over up to 1 mm. in embryonic fields of cells (Durston et al., 2011; Almirantis et al., 2013). Collinearity of different Hox clusters in the same cell and of different cells in tissues showing global collinearity is coordinated or synchronized as appropriate. Without this coordination, collinearity would not be observable. In addition to the nanoscale collinearity mechanism above, there must be a macroscale coordinating mechanism. This level of control is now proposed here as a phenomenological concept. Ideas about the molecular mechanism follow below. This way of presenting the material has the advantage that concepts are presented sequentially so this is simpler to follow.

It is interesting to ask: what is the mechanistic basis of Hox collinearity? Is nanoscale or macroscale collinearity dominant? Clearly, macroscale collinearity is dominant. This permits observation of nanocollinearity phenomena in fields of cells. There are two possibilities. The first, that the macroscale mechanism is independent of the nanoscale- and simply obliterates nanoscale collinearity- as would be the case if macroscale collinearity were regulated via sequential thresholds on a concentration gradient of a morphogen (Almirantis et al., 2013). The second, that the two mechanisms interact and macroscale collinearity drives nanoscale collinearity. This is clearly the case because nanoscale collinearity phenomena are visible at the macroscale level.

### A Macroscale Coordinating Mechanism

**FIGURE 1:**
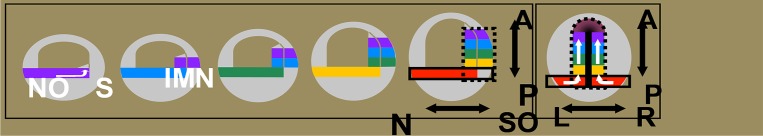
The time space translation hypothesis. Timed interactions between the Hox expressing non-organizer mesoderm (NO/N) and the Spemann organizer (S/SO) generate positional information during vertebrate gastrulation (Wacker et al., 2004; Jansen et al., 2007). Expression of new Hox genes (different colors) is initiated in non-organizer mesoderm (NO) at different times. Non-organizer mesodermal tissue moves toward the Spemann organizer by convergence and then extends anteriorly (arrow). When mesoderm adjacent to the Spemann organizer involutes (lM), the current Hox code is transferred to overlying neurectoderm (N). While the early Hox sequence in the non-organizer mesoderm (solid outlined black box) is running, new cells from this region are continuously moved into the range of Spemann organizer (dashed black box) and their Hox code is then stabilized by an organizer signal. Thus, the temporal Hox sequence is converted into a spatial AP pattern by continuous morphogenetic movement and stabilization of timed information by the organizer in both involuted mesoderm (IM) and overlying neurectoderm (N). The SO is shown only in the last drawing, as the heavy median black line. By this stage, it has become the notochord and a head mesodermal portion. The first five drawings represent paraxial profiles, where the organizer is not visible. The last drawing shows the dorsal side. The black dotted line in the last drawing depicts the sphere of influence of the SO. N, neurectoderm, NO/N, non-organizer mesoderm; S/SO, Spemann organizer; A, Anterior; P, Posterior; L, Left; R, Right. The white arrows reflect directions of cell movement flow.

**FIGURE 2:**
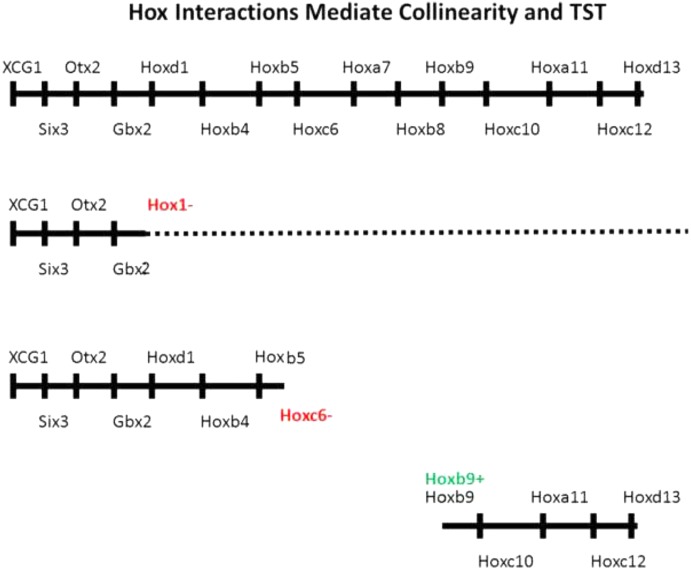
Evidence for importance of Hox–Hox interactions in collinearity. Above: Whole A-P axis with EAD genes, head genes and Hox genes. Next: Hox paralog group 1 loss of function phenotype. Expression of all genes including and posterior to Hox1 is compromised (McNulty et al., 2005). The dotted line indicates some genes have a low level of residual expression. Next: Hoxc6 loss of function phenotype. All genes including and posterior to posterior to Hoxc6 have their expression deleted or strongly reduced (Zhu et al., 2017b). Next Hoxb9 gain of function phenotype. A partial posterior axis is induced, starting at Hoxb9 following ectopic expression of Hoxb9 in a hox free dorsalized embryo. Ectopic expression of Hoxd1, Hoxb4, and Hoxb7 each generated comparable partial posterior axes, starting with Hoxd1, Hoxb4, and Hoxb7, respectively (Zhu et al., 2017a).

**FIGURE 3:**
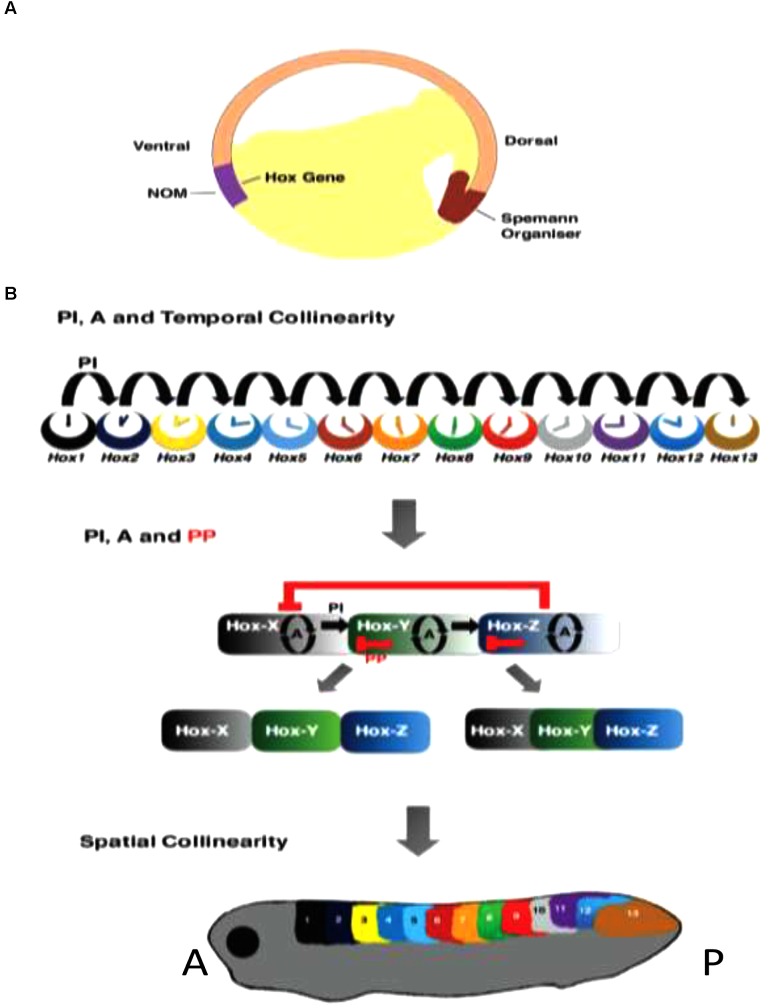
Roles of Hox–Hox interactions in temporal and spatial collinearity and TST. **(A)** Cross section of Amphibian gastrula, showing NOM and SO. **(B)** Different stages in TST. Above: Posterior induction and autoregulation (PI and A) are involved in generating temporal collinearity (Zhu et al., 2017a). Middle: the idea that later additional involvement of posterior dominnce/posterior prevalence (PD/PP) may lead to genesis of Hox zones and spatial collinearity by diminishing overlaps in expression and function between successive Hox genes. This is one concept for TST (Zhu et al., 2017a). Below: the spatially collinear pattern.

A BMP (bone morphogenetic protein- an important ventral signaling pathway in embryogenesis)-anti BMP dependent dorsoventral (D-V) mechanism generates the A-P pattern of the vertebrate main body axis by time space translation (Wacker et al., 2004; Durston and Zhu, 2015). Hox temporal collinearity generates spatial collinearity via progressive interaction of BMP dependent Hox temporally collinear NOM (non-organizer mesoderm) with anti BMP organizer signals during convergence and extension in Xenopus gastrulation. This process involves continued Hox expression in involuted, dorsalized NOM and copying of this NOM mesodermal Hox expression to the overlying dorsal neurectoderm by vertical signaling during neural transformation. It is not clear whether time space translation is mediated by copying Hox expression from temporally collinear dorsalized NOM to stably expressing neurectoderm or whether NOM Hox expression has already been stabilized during mesoderm dorsalization (**Figure 1**, **Box 1**). We show that this mechanism is mediated by three non-cell autonomous Hox–Hox interactions that have specific roles in generating the vertebrate body plan (**Figures 2**, **3**).

**BOX 1. Evidence for BMP-anti BMP dependent, Hox collinearity dependent time space translation in the Xenopus gastrula embryo.**

(1) A ventralized embryo makes no A-P axis. Just a mass of ventral tissue. Adding a Spemann organizer to a ventralized embryo causes it to make an A-P axis. One organizer: one axis, two organizers two axes etc. (Wacker et al., 2004, Durston and Zhu, 2015).
(2) Adding a Spemann organizer to a ventralized embryo at different time intervals after the beginning of gastrulation causes rescue of different parts of the A-P axis. The earlier SO is added the bigger the axial section rescued and the more anteriorly it starts. All sections run to the tail. Adding the Spemann organizer anti BMP signal noggin protein to the blastocoel of a VE at different times after the beginning of gastrulation rescues different zones in the axis. Early on: head zones including Otx2., Later: Hoxd1 Later: Krox20, later still: multiple zones representing more and more posterior sections of the axis. The age of the organizer has no effect (Wacker et al., 2004; Durston and Zhu, 2015).
(3) These results indicate an A-P timer in the ventralized embryo that imposes incrementally more posterior positional values at incrementally later times and a fixation mechanism, imposed by the Spemann organizer, that can stabilize the current positional value.
(4) The timer is in NOM (non-organizer mesoderm). The classical literature shows that mesoderm sets up the A-P pattern by progressively transmitting more and more posterior information to neurectoderm (activation-transformation) (Nieuwkoop, 1952; Eyal-Giladi, 1954). The literature shows that trunk A-P information (transformation) comes from non-organizer mesoderm (NOM) whereas neutralization signals that induce neurectoderm (activation) come from the organizer (SO). In line with this, NOM is required, together with SO and ectoderm in wrap recombinates for the ectoderm to be able to adopt a positional value (express a Hox gene). The organizer (SO) plays no role in timing. Changing the age of the SO has no effect.
(5) Hox genes are involved in determining A-P identity in the trunk. Evidence: Hox loss of function (LOF) and gain of function (GOF) phenotypes. Loss of function of a Drosophila Hox gene or vertebrate Hox paralog group (pg) dramatically changes identity of part of the axis. This effect is not necessarily restricted to a small part of the axis. For example: in Xenopus Hox1pg LOF. This downregulates all posterior Hox genes as well as Hox1. Hox1 is thus necessary for collinearity. Presumably due to interactions between Hox genes (see **Figures 2**, **3**).
(6) Hox genes show collinearity: Congruence of spatial and temporal early expression sequences with genomic sequence in Hox clusters (4 clusters in vertebrates, 1 in Drosophila). In vertebrates, spatial collinearity is first seen in neurectoderm and dorsal paraxial mesoderm), from end gastrulation (St. 12 in Xenopus). Temporal collinearity is seen earlier (mid-gastrula: St. 10.5, and through gastrulation, in non-organizer mesoderm (NOM). Changes in the Hox expression pattern during gastrulation indicate that the later spatially collinear pattern is generated from earlier temporal collinearity, as shown in **Figure 1**. Part of the NOM becomes paraxial mesoderm following its dorsalization via convergence extension movements during involution. Zones of NOM/paraxial mesoderm PM are then apparently frozen at their current involuted AP identities in an early anterior to late posterior sequence. The spatial sequence of NOM/PM zones becomes mirrored by an identical sequence of zones in the neighboring NE (in the dorsal outer wall of the late gastrula) (Wacker et al., 2004).
(7) Ventralized (UV) embryos have only NOM mesoderm (non-organizer) and have the time space translation timer and normal Hox temporal collinearity in their NOM. They have no stable Hox expression and no spatial collinearity. Their temporal collinearity is transient and later ventralized embryos have no Hox expression. Dorsalized (Li+) embryos have no NOM mesoderm (only SO) and no timer and no Hox expression (early or late). They have SO mesoderm and NE. Adding an organizer to a ventralized embryo generates an axial pattern of stable Hox expression. Spatial collinearity thus correlates 100% with the time space translation axial pattern., Temporal collinearity correlates 100% with the time space translation timer.
(8) In wrap recombinates, non-organizer mesoderm (NOM), Spemann organizer mesoderm and animal cap ectoderm (are required to enable expression of Hox genes. These markers are then most strongly expressed in the neurectoderm that develops in the recombinate. Neurectoderm but not embryonic ectoderm can express Hox genes. Omitting any of the above tissues blocks Hox expression. These recombinates mimic the *in vivo* situation. Embryonic ectoderm is the tissue from which neurectoderm is induced. The Spemnn organizer provides the signals that induce it (neutralization). The Hox expressing NOM then induces Hox expression in neurectoderm (Wacker et al., 2004).
(9) The (trunk limited) Hox TC sequence is part of a wider pan axial anti-BMP dependent timing sequence There is congruence between results on anterior TST (Tucker et al., 2008; Hashiguchi and Mullins, 2013) and our Hox results (Wacker et al., 2004). There is thus an integrated time space translation (TST) mechanism for the entire A-P axis (not confined to Hox clusters) (Durston, 2015). Conclusion: Axial patterning of the trunk in Xenopus is mediated by BMP/antiBMP dependent TST. The results above also indicate that axial TST is mediated by Hox genes in the trunk. The timer appears to be BMP dependent Hox temporal collinearity. The trunk’s A-P sequence is based on BMP inhibited Hox spatial collinearity. The trunk’s temporal collinearity generates spatial collinearity during gastrulation. TST is thus mediated by Hox genes. It is not upstream of them.

(A) Non-cell autonomous Hox autoregulation (of Hox genes and their paralogs) mediates vertical transfer of positional information from dorsalized gastrula NOM mesoderm to overlying neurectoderm- neural transformation; part of the vertebrate axial patterning process (Nieuwkoop, 1952; Eyal-Giladi, 1954; Bardine et al., 2014). This is important in, and possibly entirely mediates time space translation. Autoregulation is also clearly important in other aspects of the patterning process, e.g., mesodermal temporal collinearity (Zhu et al., 2017a) The fact that autoregulation clearly occurs under both BMP and BMP inhibited conditions suggests the possibility that it may be BMP independent.

(B) Posterior Induction- Posterior Hox genes are collinearly induced by more anterior Hox genes- at least sometimes specifically by their anterior nearest neighbors (Faiella et al., 1994; Hooiveld et al., 1999; McNulty et al., 2005; Zhu et al., 2017a,b). This interaction starts in NOM mesoderm early in gastrulation. It mediates Hox temporal collinearity.

(C) Posterior Dominance – suppression of the expression and/or function of anterior Hox genes by more posterior ones and by more posterior Hox associated micro RNA’s (Hooiveld et al., 1999; McNulty et al., 2005; Woltering and Durston, 2008; Yekta et al., 2008; Zhu et al., 2017a,b) starts only after gastrulation. It evidently plays a role in time-space translation. Posterior dominance includes, but is more than, a related function: posterior prevalence (Duboule, 1991; Duboule and Morata, 1994) that has previously been proposed. Posterior prevalence applies only to vertebrates and only to functional interactions working downstream of Hox expression. All of the instances of posterior dominance listed above regulate mRNA abundance as well as function.

Other investigations have extensively shown the importance of the same Hox–Hox interactions as above in mediating both later developmental processes and Hox expression in teratocarcinoma cells (Faiella et al., 1994; Pöpperl et al., 1995; Maconochie et al., 1997; Manzanares et al., 2001; Sheth et al., 2014).

The role of collinear Hox–Hox interactions (B,C) in generating collinearities is evident because loss of function for an appropriate Hox gene or paralog group can cut off the A-P axis or a temporally collinear Hox sequence (at the anterior expression boundary of) the gene or group in question (Faiella et al., 1994; McNulty et al., 2005; Zhu et al., 2017b) and because gain of function -ectopic expression of a particular Hox gene -initiates a collinear partial A-P axis starting at the anterior boundary of the expression zone of the ectopically expressed gene (Hooiveld et al., 1999; Zhu et al., 2017a) as well as an earlier temporally collinear Hox expression sequence in NOM mesoderm, – starting with the gene in question (**Figures 2**, **3**). Hox associated micro-RNA’s: Mir 10 and Mir 196 participate in the interactive process (Woltering and Durston, 2008; Yekta et al., 2008). These appear to exert posterior dominance but not posterior induction or autoregulation.

What, then, is the role of BMP in this mechanism? How can it promote temporal collinearity, while inhibiting spatial collinearity and neural transformation? Clearly, BMP promotes (possibly co-mediates) non-cell autonomous posterior induction. It inhibits posterior dominance and presumably vertical signaling related autoregulation. Perhaps BMP/anti-BMP can switch one Hox–Hox interaction to another. (It is known that initial temporal collinearity occurs in a BMP rich tissue (NOM mesoderm), in the early gastrula (Wacker et al., 2004). It is also well known that BMP (Dpp-decapentaplegic) acts as a cofactor for Homeoprotein mediated intercellular signaling (Layalle et al., 2011). Perhaps this is relevant for posterior induction. Posterior dominance, and the type of autoregulation that mediates “vertical signaling”- copying of Hox information from dorsalized NOM mesoderm (now dorsal paraxial mesoderm) to dorsal neurectoderm, are both inhibited by BMP (encouraged by anti-BMP). These two types of signaling are both part of the “time-space- translation” mechanism and are caused by the Spemann organizer later in gastrulation. Perhaps BMP- anti BMP switches one type of Hox–Hox interaction to another). For further discussion of signaling pathways and Hox–Hox interactions, see below (What does Hox collinearity have to do with A-P morphogens?).

### What Is the Role of Cell Movement Control?

Interesting studies in the early chicken embryo show that ectopic expression of a Hox gene early in gastrulation causes the Hox expressing epiblast cells to modulate their ingression time-thus the time they enter the primitive streak and the position where they end up on the A-P axis as if these features were regulated by Hox expression (Iimura and Pourquié, 2006). Thus, their Hox expression may determine what time cells ingress during gastrulation and where they migrate to. This movement control could in principle mediate time space translation. We don’t think it does this on its own because time space translation involves stabilization of Hox codes (Wacker et al., 2004; Dias et al., 2014) (and see above), and because it also involves neural transformation/vertical signaling (Bardine et al., 2014) (and see above) as well as specific Hox–Hox interactions (see also above). The evidence for Hox control of cell movement is interesting. It does not conflict with a role of Hox–Hox interactions in collinearity and patterning. We suggest that both contribute to making the A-P pattern.

## A Macroscale Control Mechanism for the Head

**FIGURE 4:**
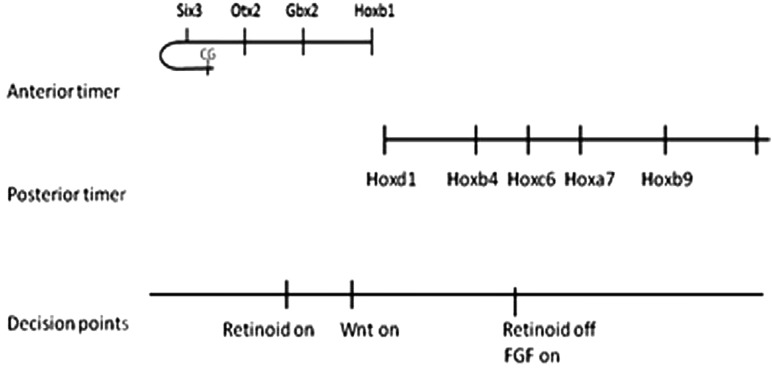
The total picture. Taken together, the different findings provide a time–space translation (TST) mechanism for the whole A-P main body axis. The most anterior part of the dorsal axis (EAD) curls around like the handle of a walking stick to face backwards on the ventral side of the embryo. The CG (Cement gland), a structure formed in this region is so far the earliest induced marker. These findings correlate with decision points in time and space where the traditionally A-P Wnt, retinoid and FGF pathways act. These decision points account for at least some of the well known actions of these pathways on A-P patterning.

Does Hox collinearity control apply only to Hox genes or does a similar mechanism also apply to determinants in the most anterior Hox free part of the axis? Hox genes are expressed and function in the axis from in the neck back to the tip of the tail. Other genes pattern the head and EAD (extreme anterior domain). The macro-level of Hox collinearity control is not restricted to Hox genes. Markers for different levels in the Hox free head domain are activated A to P, early to late sequentially by timed anti BMP pulses given at different times in the blastula-beginning gastrula stages before the earliest/most anterior Hox activation starts at mid gastrulation (Tucker et al., 2008; Hashiguchi and Mullins, 2013; Zhu et al., unpublished) The sequence of head markers is strictly A/early to P/late, just as with the Hox genes and it is A/early-P/late continuous with the Hox sequence. In addition, one marker tested for the most anterior EAD domain in the A/P axis (the cement gland marker, Xcg-1) was induced, as expected, by a pulse even earlier than various of those needed to induce head genes (Zhu et al., unpublished). We infer that these early, head inducing anti BMP signals act sequentially on a BMP dependent timer, just as with Hox genes later and that Hox-type macro collinearity control extends anteriorly into the head and EAD domains, while the nano control level is clearly Hox cluster specific. This is the final piece of evidence for a two-tier collinearity mechanism with individually regulated tiers (**Figure 4**).

## What Does Hox Collinearity Have to Do With A-P Morphogens?

Early ectopic expression of the BMP antagonist noggin causes shifts in A-P identity because this dorsalizes the axis and fixes early identities (Harland, 2000). This is logical because noggin is expected to interact with the time space translation mechanism above. In fact, we suspect it is a part of it and that BMP and/or anti BMP cooperate in one or more non-cell autonomous Hox–Hox interactions. It has been shown previously that Dpp (decapentaplegic = BMP) cooperates in a homeoprotein mediated interaction (Layalle et al., 2011).

Different morphogens and signaling pathways have been proposed to have a role in A-P patterning. Notably, retinoids (active vitamin A derivatives), FGFs (fibroblast growth factors) and Wnts (Wingless-Int growth factors). All of these molecules are well known signaling factors in embryogenesis. They and indeed the Hox genes themselves, could of course act independently of the mechanism above which is active in the very first stages of A-P patterning and they may thus later refine or remodel the primary pattern. On the other hand, they clearly do also play a part in relation to the time space translation mechanism above. Different A-P morphogens act in concert with interactions between Hox genes or between anterior determinants or between these and Hox genes to initiate and generate and delimit different axial domains (EAD, head, neck, thorax, abdomen, tail) on the A-P axis. In at least some cases, they act at or from “decision points” (Durston, 2015), situated between sequential domains where they activate expression of the first determinant in a domain and this determinant induces others by time space translation. Decision points, like expression of Hox genes, are clearly regulated in space and time.

Many findings point to the importance of retinoids in regulating the head-neck boundary on the A-P axis. For example, retinoic acid, which inhibits development of the head, collinearly induces Hox genes and initiates the posterior axis in many vertebrate embryos and in embryos of the related chordate Amphioxus. In Amphioxus, retinoid gain and loss of function have been shown, respectively, to anteriorized and posteriorize expression of the entire collinear sequence of Amphi-Hox genes and retinoids are found to act via Amphi-Hox1 which has a loss of function phenotype closely similar to that of retinoid loss of function. These findings concern the cerebral vesicle (brain)-posterior CNS (hindbrain/spinal cord) boundary between Wnt 5 and Amphi-Hox1 in Amphioxus (Schubert et al., 2004, 2006; Koop et al., 2010). Hox1 also has a role in mediating retinoic acid induced Hox temporal collinearity in human teratocarcinoma cells (Faiella et al., 1994). In the early Xenopus embryo, function of the Hox1 paralog group is also required for activation of all more posterior Hox genes as well as Hox1 (McNulty et al., 2005). These findings all point to retinoids working via Hox1 to regulate the head-neck boundary and initiating the whole collinear Hox sequence in the A-P axis. Early retinoid action seems to act at the time space translation stage of patterning to initiate the Hox pattern in the posterior central nervous system (Lloret Vilaspasa et al., 2010).

In Xenopus, Wnt8 which again is involved in turning off head and turning on neck (Niehrs, 1999) also acts at the head- neck boundary between Otx2 and Hox1 paralogs to induce Hox1 paralogs directly in NOM mesoderm (In der Rieden et al., 2010). More posterior Hox genes are induced by Wnt8 indirectly, presumably via Hox1. In this case, Wnt8 appears to act early, in NOM mesoderm during temporal collinearity.

Whereas neck Hox genes are activated by retinoic acid and Wnt, thorax Hox genes are not, but are activated by FGF (which turns on thorax Hox genes) from a decision point between Hox5 and Hox6 (Pownall et al., 1996; Godsave et al., 1998; Bel-Vialar et al., 2002). FGF acts via Cdx to induce a variety of thorax Hox genes including Hoxc6. Because the other thoracic genes are all repressed by Hoxc6 loss of function (Zhu et al., 2017b), we suspect this inductive switch is mediated exclusively via induction and action of Hoxc6.

Besides these retinoid/Wnt and FGF mediated decision points, there are presumably others in the more anterior and posterior parts of the axis.

There is also presumably a second way that Hox genes interact with morphogens in early A-P patterning. Namely that the morphogens act as co-regulators in mediating particular non-cell autonomous Hox–Hox interactions. We think that the BMP pathway cooperates with Hox genes to mediate posterior induction in at least certain parts (anterior-mid) of the axis Abdominal Hox genes (Hox 9–12) show collinear posteriorly increasing inhibition of Wnt signaling (Denans et al., 2015). This would fit the concept that anti-Wnt signaling mediates the non-cell autonomy of posterior induction in the abdominal part of the axis.

Thus, we think that BMP either co-mediates non-cell autonomy of posterior induction over the whole axis (above) or that it only does so in the parts of the axis where posterior induction is not mediated by anti-wnt. The findings and reasoning above present a case that Hox–Hox interactions cooperating with morphogen action are involved in the very first phase of vertebrate A-P patterning.

## Conclusion: Evidence for Two Tier Hox Collinearity That Mediates the Vertebrate Bodyplan

The dominant (macro) collinearity tier and lesser (nano) collinearity tier have different domains of action. The macro mechanism extends into the head and EAD as well as through the trunk Hox expressing part of the axis. It thus covers the whole axis. The nano mechanism is specific to Hox clusters and is therefore presumably restricted to the Hox expressing part of the axis (although it cannot yet be ruled out that the anterior determinants in the axis are contained in clusters (as with the Hox and ParaHox genes) and have a parallel mechanism).

Macro collinearity operates and connects to axial patterning via Hox–Hox interactions. This tier is divided into axial domains via interactions at decision points with axial signaling pathways (see above and below). Most of this mechanism was detected and characterized initially in Xenopus. Head macrocollinearity was detected initially in zebrafish and confirmed in Xenopus (Tucker et al., 2008; Hashiguchi and Mullins, 2013; Zhu et al., unpublished). TST was discovered in Xenopus (Wacker et al., 2004) and confirmed in chicken (Dias et al., 2014). Initial temporal collinearity and its role in generating later spatial collinearity was initially hypothesized by Duboule (1994). It was shown in Xenopus (Wacker et al., 2004) but has also later been confirmed in mouse (Deschamps and Duboule, 2017).

These thoughts obviously pose many questions. Nonetheless, they rest on potentially significant recent developments concerning Hox collinearity and the bodyplan and perhaps provide a realistic framework for thinking about vertebrate axial patterning.

The findings above lead to the conclusion that Hox–Hox interactions are active in the very first phases of vertebrate A-P patterning. We used timing expts to show that Hox posterior induction and autoregulation are active from the beginning of and posterior dominance from the end of gastrulation (stages 10.5 and 12 respectively in Xenopus) (Zhu et al., 2017a).

These Hox–Hox interactions interact with signaling pathways that are known to be relevant for A-P patterning. One type of signaling pathway–Hox interaction is that a signaling pathway initiates a collinear sequence of Hox expression, starting at a specific decision point between two axial domains (as seen with retinoids at the head- neck boundary (CNS) and putatively with Wnt at the head-neck boundary (mesoderm) and putatively with FGF-cdx at the neck-thorax boundary.

A second type of interaction is that a signaling pathway cooperates with Hox genes to enable a Hox induced non-cell autonomous interaction. This is true for the BMP pathway which is required for posterior induction among at least some (the more 3′), and possibly all. Hox genes. Conversely the evidence suggests that BMP inhibits (and anti BMP promotes) posterior dominance among the same Hox genes. On the other hand, there is evidence suggesting that anti Wnt may be involved in non-cell autonomous cell interactions among 5′posterior (abdominal) Hox genes. This could be additionally to or in place of (anti-)BMP.

In conclusion, the evidence above points to a two tier collinearity mechanism, that is the basis of the earliest phase of vertebrate A-P patterning (where the pattern is laid down). This acts in the anterior head and EAD parts of the axis as well as in the posterior Hox-expressing parts and cooperates with the known A-P signaling pathways which are concerned with defining the axial domains of the pattern.

## Author Contributions

The author confirms being the sole contributor of this work and approved it for publication.

## Conflict of Interest Statement

The author declares that the research was conducted in the absence of any commercial or financial relationships that could be construed as a potential conflict of interest.
